# Supplementary figures and images for: Up-regulation of cytosolic prostaglandin E synthase in fetal-membrane and amniotic prostaglandin E2 accumulation in labor
Source: PLoS One. 2021 Apr 23;16(4):e0250638. doi: 10.1371/journal.pone.0250638 (PMC8064594; doi:10.1371/journal.pone.0250638)

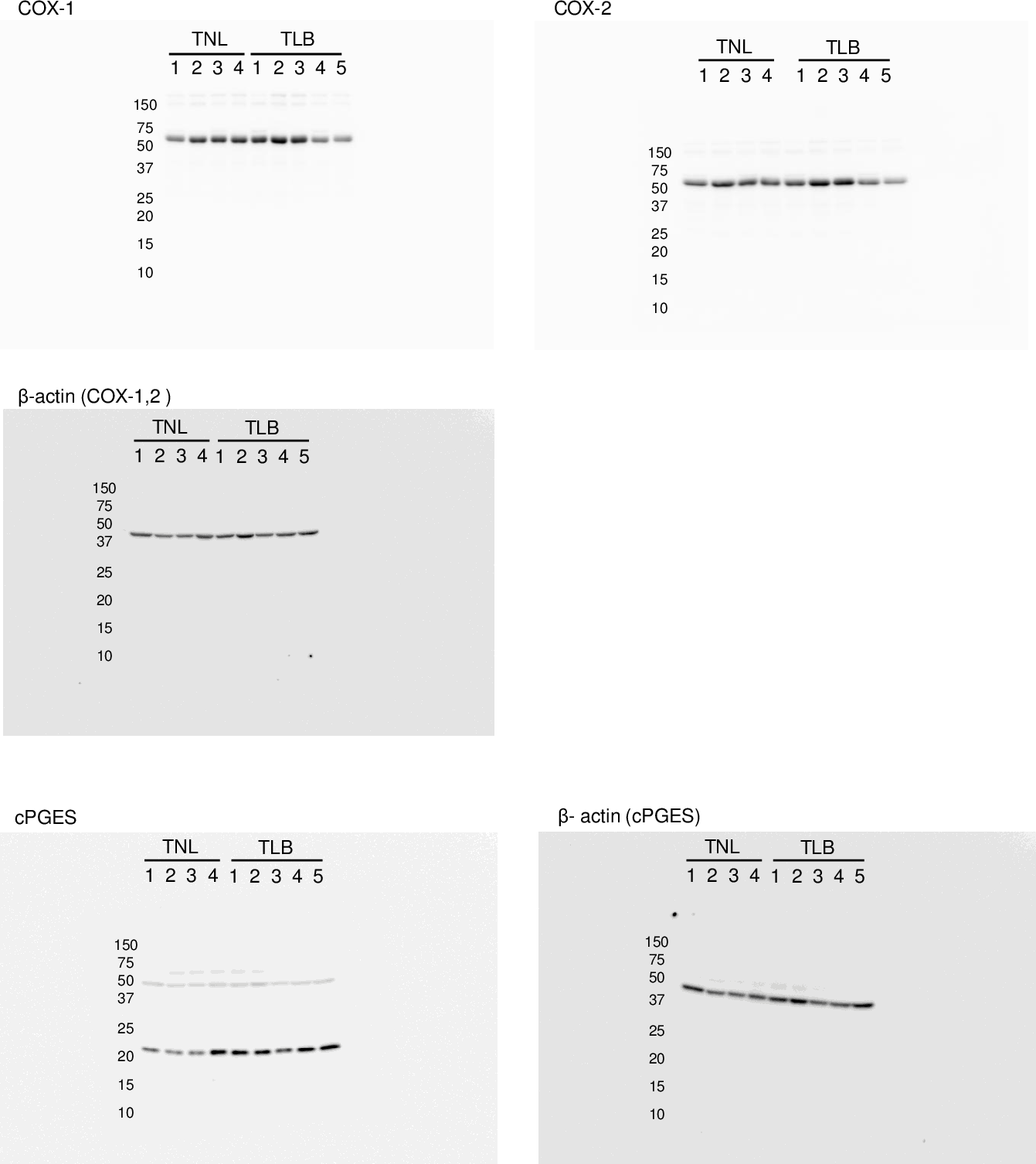

Supplement: S1 Fig — (TIF) [file pone.0250638.s001.tif]
